# Biodesulfurization of Fossil Fuels: Analysis and Prospective

**DOI:** 10.12688/f1000research.133427.1

**Published:** 2023-09-08

**Authors:** Wisam Mohammed Kareem Al-Khazaali, Seyed Ahmad Ataei, Saeed Khesareh

**Affiliations:** 1 Chemical Engineering Sec., Designs Dept., Projects Div., Misan Oil Company, Misan Governorate, Iraq; 2 Department of Chemical Engineering, Faculty of Engineering, Shahid Bahonar University of Kerman, Kerman, Iran

**Keywords:** Biodesulfurization, aerobic, anaerobic, recalcitrant organosulfur compounds.

## Abstract

Biodesulfurization (BDS) of fossil fuels is a promising method for treating the high content of sulfur in crude oils and their derivatives in the future, attributed to its environmental-friendly nature and the technical efficient ability to desulfurize the organosulfur compounds recalcitrant on other techniques. It was found that the bioreaction rate depends on the treated fluid, targeting sulfur compounds, and the microorganism applied. Also, many studies investigated the operation conditions, specificity, and biocatalysts modification to develop BDS efficiency. Furthermore, mathematical kinetics models were formulated to represent the process. In this review, the previous studies are analyzed and discussed. This review article is characterized by a clear picture of all BDS’s experimental, industrial, procedural, theoretical, and hypothetical points.

## 1. Introduction

Fossil fuel (FF) is a crucial source of energy and power in numerous industries and aspects of daily life. However, to ensure Health, Safety, and Environmental (HSE) protection, it must adhere to relevant standards before use, including the treatment of sulfur compounds. Fossil fuels come in various forms, such as coal, crude oil, tar, petroleum fractions, shale oil, and sands. Regulations for HSE, quantity assurance, and quality control (QQHSSE) are based on specific criteria, including:

• The technical quality of crude oil is determined by its specifications, including the American Petroleum Institute (API) density, viscosity, and combustion heat value. These specifications can be influenced by the presence of organic sulfur compounds in refineries, as highlighted by various studies ( Adlakha *et al*., 2016; Bergh, 2012; Sadare *et al*., 2017; Alves *et al.*, 2015).
• The sustainable quantity of crude oil is indirectly influenced by the presence of sulfur, which can limit or reduce its marketability due to its negative impact on QQHSSE criteria. As a result, many efforts are being made towards clean energy, which has led to a commitment to meeting quality standards in various regions.
• The health impact of sulfur is significant, as sulfur dioxide emissions can lead to safety hazards, corrosion, and environmental leaks that can cause heart disease, asthma, and respiratory ailments, as evidenced by various studies ( Sadare *et al*., 2017; Srivastava, 2012). Hydrogen sulfide (H _2_S) is another hazardous sulfur compound that can cause acute toxicity, leading to fatalities in natural settings and workplaces. Exposure to H _2_S can also result in loss of consciousness, paralysis, nervous system disorders, cardiovascular disorders, ocular disorders, gastrointestinal disorders, and even death, as highlighted by Rashidi *et al*. (2015).
• The safety implications of sulfur compounds are significant, as their release into the environment can lead to pollution, acid rain, and damage to buildings and forests, as noted by Sadare *et al*. (2017). Additionally, sulfur compounds can poison the catalytic converters in automotive engines, leading to premature failure and reduced efficiency, as highlighted by Sadare *et al*. (2017) and Bergh (2012). The presence of sulfur in liquid fuels can also harm vehicles and lower the efficiency of catalytic converters, as noted by Sadare *et al*. (2017) and Srivastava (2012). Furthermore, sulfur can cause corrosion of pipelines, pumps, fittings, and refining equipment, as noted by Adlakha *et al*. (2016). The poisoning or deactivation of catalysts used in refining is another concern, as higher levels of sulfur in petroleum distillates can lead to the deactivation of catalysts by poisoning the fluid catalytic cracking (FCC) and hydrocracking processes that convert heavy distillates to lighter ones, as highlighted by Sadare *et al*. (2017).
• Environment: The combustion of fossil fuels can result in the release of harmful components such as SO _x_, NO _x_, CO _2_, and H _2_S, which can have serious environmental consequences. These emissions can cause acid rain, acid deposition, and severe air pollution, which can be detrimental to agriculture, human health, and wildlife ( Chen *et al*., 2019a; Sadare *et al*., 2017; Srivastava, 2012; Ansari, 2017; Pacheco *et al*., 2019).
• As a result of these concerns and in order to implement risk control measures, the Clean Air Act of 1964 was introduced, with subsequent amendments in 1990 and 1999. These laws were strengthened with even more stringent requirements aimed at further reducing the quantity of sulfur released into the air. Currently, the U.S. Environmental Protection Agency (EPA) has revised the sulfur standards to the following levels:
• The current standard of 500 ppmw for diesel fuel was replaced with a stricter requirement of 15 ppmw, which became effective in mid-2006.
• The standard for reformulated gasoline has been revised to lower the limit from 300 ppmw to 80 ppmw, with a yearly average not exceeding 10 ppm, effective from January 1, 2004.

Similarly, the EU has also implemented similar modifications by setting a sulfur limit of 10 ppmw for diesel fuels and gasoline in 2005 to address these adverse effects. In order to mitigate these effects, environmental agencies in various countries around the world have imposed more stringent legislative provisions on the total sulfur content of oil ( Chen *et al*., 2018, 2019a, 2019b).

This review provides a more comprehensive analysis of modern chemicals and biotechnology compared to previous review papers. Additionally, the study offers a significant insight into the modeling and optimization of biodesulfurization.

## 2. Desulfurization methods

There exist various treatment methods that can be classified into three categories: physical, chemical, or biological. These methods include lab or industrial techniques, such as adsorptive desulfurization (ADS) ( Akhmadullin *et al*., 2012; Al-Otaibi., 2013; Dantas *et al*., 2014; Mujahid *et al*., 2020; Sadare *et al*., 2017), alkylation-based desulfurization, microbial or bacterial desulfurization (BDS) (Chen *et al*., 2019), catalytic reaction treatment ( Akhmadullin *et al*., 2012; Jarullah, 2012), sodium metal reaction ( Chavan *et al*., 2012), Caustic Washing Method for demercaptanization and H _2_S removal, Chlorinolysis-based desulfurization, Extraction (desulfurization by extraction, EDS) ( Heidari *et al*., 2013; Qader *et al*., 2021), dry gas desulfurization, hydrodesulfurization (HDS), oxidative desulfurization (ODS) ( Sadare *et al*., 2017), and supercritical water-based desulfurization (SWD) ( Siddiqui and Ahmed, 2016).

Compared to other methods that are not yet commercialized, BDS is capable of removing refractory heterocyclic compounds in crude oil to achieve ultra-low sulfur diesel (ULSD) with high efficiency and at an ultra-low cost ( Malani *et al*., 2021). Undoubtedly, BDS is suitable for desulfurizing heavy oils, such as shale oils, which have higher concentrations of thiophene ( Mohebali and Ball, 2016; Pacheco *et al*., 2019), making it a promising method for commercialization. Moreover, it is more efficient and cost-effective than HDS.

Harsh reaction conditions are required for the hydrodesulfurization (HDS) process to remove certain recalcitrant sulfur-containing compounds like alkylated dibenzothiophenes (DBTs), which can lead to fuel degradation and reduced quality. In contrast, bacterial desulfurization (BDS) has shown high efficiency and low cost for ultra-low sulfur diesel (ULSD) production and is a promising alternative to HDS. Therefore, several studies have been conducted to facilitate the industrial application of BDS due to its advantages over HDS ( Chen *et al*., 2019b; Mohebali and Ball, 2016; Sadare *et al*., 2017).

Some examples of combined desulfurization methods are BDS-ODS-EDS ( Duissenov, 2013), EDS-HDT ( Hamad *et al*., 2013), OEDS (oxidation extraction desulfurization) ( Awad, 2015; Jiang *et al*., 2018; Sadare *et al*., 2017), and the microwave catalytic hydrogenation process ( Duissenov, 2013; El-Gendy *et al*., 2014).

## 3. Susceptibility of treated oils and biodesulfurizers

BDS is capable of treating various pretreated crude oils and its derivatives, including LPG, gasoline, jet fuel, kerosene, fuel oil, and gas oil. HCS may be present in these fractions or the entire crude oil, including alkyl sulfides (thioesters), carbon bisulfide, disulfides, hydro thiophene, mercaptans (thiol “alkyl hydrosulphide” and thiophenol), thiophenes, thiophane, thiolates, sulfides (non-cyclic sulfides, cyclic sulfides), sulfoxides, sulfones, and sulfonic acid.

Few studies and patents have been conducted on the application of *Aspergillus flavus*, *Achromobacter* Spp., *Leptospirillum* Spp., *Pseudomonas* Spp., *Sulfolobus* Spp., *Thiobacillus* Spp *.*, *Rhodococcus* Spp., *Sphingomonas subarctica*, *Bacillus* Spp., *Desulfovibrio desulfuricans*, *Pyrococcus* Spp., *Desulfomicrobium scambium*, *Desulfovibrio longreachii*, or *Pantoea agglomerans* for the desulfurization of whole crude oil under aerobic or anaerobic conditions ( Adegunlola *et al*., 2012; Gunam *et al*., 2013; Yang *et al*., 2016). The advantage of BDS for the desulfurization of whole crude oil is that it can reduce the cost of the desulfurization treatment in refineries ( El-Gendy and Nassar, 2018; Al-Jailawi *et al.*, 2015).

In addition, microorganisms such as *Mycobacterium goodii*, *Pseudomonas* Spp., *Gordonia* Spp., *Rhodococcus* Spp., *Mycobacterium phlei*, *Paenibacillus* Spp., *Rhodococcus globerulus*, and *Nocardia* Spp. have also been used for BDS treatment of gas oil, gasoline, petro-diesel fuels, petroleum wastes, fuel oil, and cracked stocks ( El-Gendy and Nassar, 2018; Li and Jiang, 2013; Murarka and Srivastava, 2020; Nassar *et al*., 2021a).

In addition, model compounds representing the recalcitrant HCS in fossil fuels can be used to study the efficiency of BDS. These compounds can be in the form of pure or mixture solutions such as B.T., DBT, DBTO2, M DBTSO2, MgSO4, BNT, DBS, 2,8 DMDBT, 2,6 DNDBT, DMDBT, thianthrene, and dibenzyl sulfide ( Alejandro *et al*., 2014; Boshagh *et al*., 2014; Bordoloi *et al*., 2014; Jiang *et al*., 2014; Kawaguchi *et al*., 2012; Nassar *et al*., 2013; Sohrabi *et al*., 2012; Zhang *et al*., 2013). It is worth noting that microorganisms used for BDS on water or coal could also be effective for crude oil applications ( Feng *et al*., 2018). A recent study conducted in 2022 by Al-Kazaali ( Al-Kazaali and Ataei, 2022) investigated the BDS of heavy sour crude oil using various microorganisms and media. Table 1 summarizes the recent research on BDS application on various fractions of crude oil using different microorganisms.

**Table 1.** Last Recent treatment case studies of real and model fractions.

Fraction	Microorganism	Conditions	Response	Reference
Coal	*Acidithiobacillus* ferrooxidans LY01 domesticated with ferrous iron and pyrite	28°C 180 rpm 3 d	67.8% at case: pyrite 45.6% at the case: Fe (II)	( Yang *et al*., 2016)
DBT 0.3 mM	*Paenibacillus* PO-2 Basal salt medium, sulfure-free	30°C 180 rpm 3 d	95%	( Derikvand and Etemadifar, 2015)
Crude oil light Cs=1.5%	*Bacillus subtilis* Wb600 Incubator/shaker/thermos	T 308 K 150 rpm t 90 h	40%	( Nezammahalleh, 2015)
Crude oil, gas oil	*Gordonia* sp. IITR100	30°C 180 rpm 7 d	76.1% at the case: HCO 9.8% at case: gas oil-1 70% at the case: gas oil-2	( Adlakha *et al*., 2016)
B.T., DBT, MDBT, DMDBT, DBS	*Stenotrophomonas* isolates AK9 Chemical-defined medium, sulfur-free	30°C 180 rpm 4 h	90% DBT	( Ismail *et al*., 2016)
Crude oil, gas oil, kerosene,benzen, DBT,DBTO2,MgS4	*Rhodococcus Pseudomonas Bacillus*	30°C 200 rpm 15 d	30%	( Shahaby and Essam-El-din, 2017)
DBT 0.1 mM MgSO4: 0.2 mM	*Ralstonia eutropha* strain FMF Basal Salt medium, pH 7	30°C 150 rpm 24 hr	20%	( Dejaloud *et al*., 2017)
Wastewater	*Halothiobacillus* neapolitanus Basal Salt medium	30°C 170 rpm 100 hr	85%	( Feng *et al*., 2018)
DBT	*Gordonia alkanivorans* strain 1B			( Pacheco *et al*., 2019)
DBT	*Gordonia* sp. SC-10 Sulfur-free medium	30°C 160 rpm 5 d	81%	( Chen *et al*., 2019a)
Gas oil	*Gordonia* sp. SC-10 Sulfur-free medium	30°C 160 rpm 5 days	80% at case: OWR 1/12	( Chen *et al*., 2019b)
DBT	*Ralstonia eutropha* PTCC 1615	pH 6-9	98% at case: pH 8	( Dejaloud *et al*., 2019)
B.T., DBT, and derivatives	*Klebsiella Pseudomonas Rhodococcus Sphingobacterium* Chemical-defined medium	180 rpm	25 %	( Awadh *et al*., 2020)
Gas oil	*Paenibacillus* glucanolyticus HN4 Basal Salt medium	30°C 150 rpm	<80%	( Nassar *et al*., 2021a)
DBT and derivatives	*Gordonia alkanivorans* strain 1B BSM-SF	30°C 150 rpm 14 hr	average q2-HBP, 2-HBP specific production rate (μmol/g (DCW): 1.5	( Silva *et al*., 2020)
Gas oil 169 mg/l DBT, MDBT, DMDBT	*Gordonia* sp. SC-10 SFM	30°C 160 rpm 4 d	88%	( Chen *et al*., 2021)
Gas oil 280 mg/l, BT, DBT, DBDBT, DHDBT	*Sphingomonas subarctica* T7b MSSFCA medium was	27°C 273 rpm 5.5 d	41.4% at case: gas oil 82% at the case: DBT 80% at the case: DBDBT	( Gunam *et al*., 2021b)
Crude oil 0.4%	*Pseudomonas aeruginosa* NB	30°C	50%	( Saeed *et al*., 2022)
Crude oil 4%	*Ralstonia eutropha Rhodococcus erythropolis Acidithiobacillus ferrooxidans Acidithiobacillus thiooxidans*	30-50°C	>80%	( Al-Kazaali and Ataei, 2022)

Desulfurizing microorganisms can be classified based on their air requirement ( Raheb, 2016) into the following categories:

• Aerobic conditions
• Anaerobic conditions
• Favored anaerobic

### 3.1 Aerobic conditions

The reaction mixture for biodesulfurization contains the fluid to be treated, which could be crude oil, one of its derivatives, or model compounds of the recalcitrant HCS such as benzothiophene (BT) or DBT. This fluid is mixed with the main nutrient medium, trace element solution, and vitamin solution. The microorganism is then added to this reaction mixture. The equipment used for BDS can be either a simple incubator or an airlift reactor.

*Rhodococcus* strains have been widely used in BDS to desulfurize various fractions, including pure DBT and its alkylated derivatives such as 4,6-DB-DBT and 4-HDBT, as well as light gas oil by *Sphingomonas subarctica* T7b ( Mohamed *et al*., 2015). Other microorganisms that have been applied in BDS for real fluids and model compounds include *Bacillus* strains such as subtilis DSMZ 3256, *Gordonia* strains such as IITR 100 and *D. alkanivorans* strain 1B, *Klebsiella* sp. 13T, *Mycobacterium goodii* X7B, *Pseudomonas* strains such as *P. delafieldii* R-8, *P. aeruginosa*, and *P. putida* CECT5279, *Paenibacillus validus* strain PD2, and *Stachybotrys* sp. ( AL-Faraas *et al*., 2015; Boshagh *et al*., 2014; Bhatia and Sharma, 2012; Chauhan *et al*., 2015; Derikvand and Etemadifar, 2015). Moreover, the BDS of TH, BTH, or DBT by sulfur-reducing bacteria (SRB) has been studied, as well as coal and pyrite by *Theobacillus ferroxidans* and *Ascidians* Spp. Brierley ( Rossi, 2014).

### 3.2 Anaerobic conditions

Anaerobic BDS has been reported for crude oil and its distillates using microorganisms such as *Desulfovibrio desulfuricans* M6 and extreme thermophile *Pyrococcus furiosus.* In addition, BDS of thiophene, benzothiophene, and benzothiophene were detected in anaerobic mixed microbial communities of SRB from three Russian oilfields. These communities used reducing processes and produced sulfide-containing hydrogen, lactate, and ethanol as potential electron donors. The conversion of DBT to biphenyl was observed, but HC products from BTH and TH desulfurization remained undetected. Further enrichment of thiophene as the only electron acceptor led to the disappearance of conversion activity, and homo-acetogenic bacteria were abundantly present. Attempts were made to isolate sulfide-producing bacteria to attain stable conversions of THs, but the activity remained low, and homo-acetogenesis was dominant. However, the rate of anaerobic BDS is low, which prevents commercialization.

### 3.3 Favored anaerobic

Many aerobic microorganisms have been studied for crude oil BDS, including *Pantoea agglomerans* D23W3 and *Klebsiella* sp. 13T. Additionally, biotreatment can be classified based on the temperature conditions into thermophilic and mesophilic microorganisms.

## 4. Biodesulfurization performance

There are many factors that can affect the efficiency of BDS treatment. Physical factors include operation conditions, such as pressure, temperature, time, and mixing rate. Chemical factors include the medium used to create an appropriate environment for the microorganisms. Biological factors are represented by the presence and concentration of the added microorganisms, which can be either thermophilic or mesophilic, such as *Paenibacillus* Spp. and *Rhodococcus erythropolis*, respectively. Microorganisms can also be used under either aerobic or anaerobic conditions, such as *Gordonia* IITR 100 and *Desulfovibrio desulfuricans* M6, respectively. Moreover, microorganisms can be used in different forms, including pure or single microorganisms, multiple bacterial bio-mixtures with known presence ratios, and isolated colonies. It is important to note that certain bacterial isolates, such as *Rhodococcus* Spp., have shown a particular ability to attack heterocyclic HCSs and desulfurize derivatives of thiophene found in petroleum.

Given this objective, and given that the physical and chemical factors serve to support the biological factors, the biological factors can be considered as the primary treatment agents, and the process is referred to as biotreatment, or BDS. As a result, the BDS treatment is carried out under the influence of biotreaters.

Another important factor to consider in BDS treatment is aeration. Aerobic microorganisms depend on the presence of oxygen for their growth and metabolism, such as *Arthrobacter simplex*, *Acidithiobacillus* Spp., and *Rhodococcus* Spp., while anaerobic microorganisms can survive in the absence of oxygen, such as SRB and *Desulfovibrio desulfuricans* M6.

The efficiency of BDS is affected by various factors such as the type of microorganism used, the level of biopurity (septic, semi-septic, or aseptic), and the environmental conditions (quantity and types of nutrients available). The differences in the ratio of sources of carbon (C), nitrogen (N), and phosphorus (P) can significantly impact the efficiency of crude oil desulfurization as they affect the growth and treatment of microorganisms. The optimal ratio of carbohydrates/nitrates/phosphates in the microorganism strain and cell volume should be considered to maximize the desulfurization efficiency.

The efficiency of BDS varies depending on the microorganism used, the biopurity status, and the environmental status. The efficiency can also be affected by the specificity of microorganisms towards different types of HCS present in fossil fuels, as well as the competition for available sources of sulfur compounds. Multiple substrates can also decrease efficiency compared to a single substrate. Microorganisms have different efficiencies for desulfurizing crude oil and its derivatives due to the presence of various HCS in different fractions. However, the pure rate of BDS is generally low. To increase the bioreaction rate, electrokinetic or sonochemical fields can be used ( Awadh *et al*., 2020; Chauhan *et al*., 2015; Gunam *et al*., 2021b; Ismail *et al*., 2016; Shahaby and Essam-El-din, 2017; Boshagh *et al*., 2014).

## 5. Isolation and identification of microorganisms

Microorganisms can be prepared and identified using various techniques. They can be isolated from soil contaminated with hydrocarbon fuel, the fuel itself, or through the cultivation of pure microorganisms obtained from culture collections such as ATCC and PTCC. Fermentation processes can then be used to cultivate the microorganisms.

The microorganisms were isolated by inoculating the samples onto suitable culture media and incubated under appropriate conditions in a shaker incubator. For the bacterial mixture, a suitable medium containing distilled water, microorganism seeds, a source of carbon and energy, and sulfur for adaptation was used. The growth environment for each isolate typically includes sources of energy such as carbon or iron sources, nitrogen, metals, and vitamins. *Rhodococcus* strains, such as *R. erythropolis*, have been isolated from contaminated soil using basal salts or sulfur-free medium (Jarullah, 2012). Other isolates, such as *Gordonia* sp., *Noocaria* sp., *Pantoea agglomerans*, *Pseudomonas delafieldii*, *Pseudomonas aeruginosa*, SRB, and *Sphingomonas subarctica*, have also been prepared and studied ( Chen *et al*., 2019a, 2019b; Gunam *et al*., 2021a, 2021b; Saeed *et al*., 2022).

There are various methods available for the identification of microorganisms based on their physiological, chemotaxonomical, chemical, and biochemical properties. Gram staining is one such method that distinguishes between gram-negative and gram-positive bacteria, while other characteristics such as metabolic types, oxygen requirements, and motility can also be used for classification. For instance, Rhodococcus Spp. can be identified based on their mycolic acid, DAP acid, and cell wall sugar composition, as well as their menaquinone profile. Other methods such as fatty acid analysis, 16S rRNA gene sequencing, and PCR amplification can also be employed for microbial identification, as exemplified by studies on Rhodococcus erythropolis and Gordonia Spp. ( Morales and Le Borgne, 2017; Bergey’s manual of systematic bacteriology).

Other resources for microbial identification include the Deutsche Sammlung von Microorganismen und Zellkulturen GmbH (DSMZ). In some cases, techniques such as electroporation can be used to improve the genetic manipulation of microorganisms, such as the genus Gordonia.

## 6. Experimental developments technics

The studies aimed to target the treatment of whole crude oil by modeling various compounds as single or mixtures of hydrocarbons (HC) and heterocyclic sulfur compounds (HCS) to represent the required treatment crude and target, especially to treat the recalcitrant HCS on hydrodesulfurization (HDS) and oxidative desulfurization (ODS).

### 6.1 Biocatalysts

Studies have focused on using biocatalysts or biotreaters as a means of treating crude oil, with a particular emphasis on targeting the recalcitrant HCS on HDS and ODS. Various improvements have been reported, including the use of recombinant strains, DNA (primers, operons, enzymes, and promoters), non-cell biocatalysts (monooxygenases, oxidases, and peroxidases), thermophilic enzymes, increasing the expression of key enzymes, expression of desulfurization enzymes in heterologous hosts, alternate cells for the expression of *dsz* genes, coexpression of *dsz* genes ( *dsz*, *tds*, *mds*, sox), increasing the number of desulfurization genes present, changes in the dsz operon’s gene order, the addition of flavin reductase, modifications to the dsz operon’s promoter, rearrangements of the dsz gene cluster, alterations to translational sequences, and the use of multiple strains or wild strains of some microorganisms. These changes have been reported in various studies ( Boniek *et al*., 2015; Calzada *et al*., 2011; Chauhan *et al*., 2015; El-Gendy and Nassar, 2018; Kawaguchi *et al*., 2012; Kilbane and Star, 2016; Li *et al*., 2019; Malani *et al*., 2021; Mohebali and Ball, 2016; Nazari *et al*., 2017; Yaqoub, 2013; Martinez *et al.*, 2016).

Modifying the expression of enzymes or the genes involved in the process can lead to biostability, which can increase the likelihood of commercialization ( Nazari *et al*., 2017). Modifying the enzymes involved in the pathway can also lead to the development of a more efficient and effective treatment process ( Raheb *et al*., 2011).

### 6.2 Systems

The efficiency of BDS can be affected by the reactor type, such as a stirred tank reactor, airlift reactor, or bubble column bioreactor, as well as the reactor size and geometry, which can be related to the flask or bioreactor. Several studies have reported on these factors, including Chen *et al*. (2018), Malani *et al*. (2021), Martínez *et al*. (2016), Prasoulas *et al*. (2021), and Zhang *et al*. (2013). Amin *et al*. (2013) designed two vertical rotating immobilized cell reactors (VRICR) and applied *Bacillus subtilis* BDCC-USA-3 and *Rhodococcus erythropolis* ATCC 53868 with bio-emulsifier in BDS of DBT.

Process design and scale-up are crucial aspects of BDS. The process package typically involves a microorganism generator or fermenter for the vegetation step, a bioreactor for the multi-stage or series process, separation of O/W/microorganism and precipitation of sulfates using lime or salts, biocatalyst recycling, and residual oil purification units ( Mohebali and Ball, 2016; Nazari *et al*., 2017; Speight and El-Gendy, 2017). BDS can be intensified by using various physical, chemical, and biochemical methods, such as ultrasonic irradiation (sonication) as a stirrer ( Tang *et al*., 2013), the application of electricity ( Malani *et al*., 2021; Yi *et al*., 2019), and a combination of units, such as microwave-catalytic hydrogenation, BDS-ODS-RA, and Ads-BDS ( Duissenov, 2013; Mohebali and Ball, 2016). Additionally, coupling BDS with other treatment methods, such as bio-hydrotreater or adsorption, can lead to better efficiency.

### 6.3 Environment

Environmental factors play an important role in biodesulfurization (BDS). The selection of an appropriate medium composition is crucial, with water being the most commonly used basis for microorganism growth. The medium should be tailored to the specific requirements of the microorganism, with sources of carbon, energy, and sulfur chosen based on their metabolic needs ( Duissenov, 2013; Pacheco *et al*., 2019). One way to enhance BDS is by controlling mass transfer limitation of oxygen and hydrogen sulfide ( Mohebali and Ball, 2016), as well as reaction rate and pathways through the use of emulsifiers ( Duissenov, 2013; Li and Jiang, 2013; Karimi *et al*., 2017; Jiang *et al*., 2014; Malani *et al*., 2021; Mohebali and Ball, 2016; Martínez *et al*., 2016; Martinez *et al*., 2015; Rocha e Silva *et al*., 2017). Immobilization techniques, such as using alginate beads or lignin nanoparticles, can also improve BDS efficiency ( Amin, 2011; Capecchi *et al*., 2020; Gunam *et al*., 2013; Gunam *et al*., 2021a, 2021b; Nassar *et al*., 2021a, 2021b; Silva *et al*., 2020). Chemical catalysts and host improvers have also been used to enhance BDS ( Malani *et al*., 2021; Silva *et al*., 2020; Nazari *et al*., 2017; Sousa *et al*., 2016). In addition, diagnostic GFP fusions can be employed to improve pathway efficiency.

### 6.4 Improvement of factors

Optimization studies in BDS often focus on identifying the best pre-treatment methods, such as isolation techniques or desulfurization conditions. For mixtures, optimization involves determining the operating conditions of BDS to achieve the desired selectivity, while for pure substrates or lumped mixtures, the aim is to determine the availability or specificity of the microorganism. Intensification can also be optimized by defining a suitable range. Several studies have investigated optimization in BDS, including Awadh *et al*. (2020), Boniek *et al*. (2015), Chen *et al*. (2019b), Chen *et al*. (2021), and Chauhan *et al*. (2015).

### 6.5 Selectivity of target sulfur compounds

One of the main objectives in biodesulfurization research is to understand the preferential attraction of each microorganism to the different types of HCS, both under aerobic and anaerobic conditions ( Awadh *et al*., 2020; Mohebali and Ball, 2016). This information can be used to optimize the selection of microorganisms for specific HCS desulfurization processes. Table 2 provides an overview of the most significant biotechnology techniques for desulfurization of hydrocarbon fuel fractions.

**Table 2.** Last modern applications of high technologies.

Fraction	Microorganism	Applied High Technology	Reference
DBT derivatives	*Pseudomonas Putida*	Co-substrates-gene	( Martinez *et al*., 2015)
DBT	*Pseudomonas Putida*	Oxygen mass transfer	( Martínez *et al*., 2016)
DBT	*Pseudomonas Putida*	dsz cassettes	( Martínez *et al*., 2016)
DBT	*Brevibacillus* brevis BSM	Mathematical modeling	( Nassar *et al*., 2016)
DBT	*Rhodococcus erythropolis*	Nanotechnology	( Karimi *et al*., 2017)
		Process Design	( Roodbari *et al*., 2016)
DBT	*E. coli*	Desensitization + Overexpression	( Li *et al*., 2019)
DBT, NT, derivatives	-	ultrasound ODS UODS	( Yi *et al*., 2019)
Gas oil	*Rhodococcus*	Immobilizer (SPION)	( Nassar *et al*., 2021b)
DBT	*Pseudomonas*	immobilizer	( Gunam *et al*., 2021a)
Crude oil	*Rhodococcus erythropolis* IGTS8	Increase the rate of limiting steps in the pathway	( Sousa *et al*., 2016)

## 7. Theoretical development methods

Theoretical development involves both mathematical modeling and statistical optimization of the process to simulate the treatment phenomena and achieve higher quality and lower costs, respectively.

### 7.1 Kinetic mechanism pathway

Microorganisms have various pathways for oxidative or reductive cleavage of C-S or C-C bonds, as shown in Table 3. However, it is advisable to selectively remove the sulfur atom from the hydrocarbon (HC) structure (such as DBT) through the 4S-pathway while retaining its HC skeleton and fuel value. This produces non-toxic or less toxic compounds such as 2-HBP, 2,2′-bihydroxybiphenyl, 2-methoxybiphenyl, and 2,2′-dimethoxy-1,1′-biphenyl, which are divided into the H.C. phase (i.e., the fuel) while the sulfur is eliminated as inorganic sulfate in the aqueous phase containing the biocatalyst ( Bhatia and Sharma, 2012; Bordoloi *et al*., 2014; El-Gendy and Nassar, 2018; Hirschler *et al*., 2021; Kilbane and Star, 2016; Malani *et al*., 2021; Mohebali and Ball, 2016).

**Table 3.** Metabolisms pathways of microorganisms.

Microorganism	Reference
Bond Specificity: Oxidative cleavage C-C bond, Pathway: Kodama pathway	
*Pseudomonas Putida*	( Malani *et al*., 2021)
Bond Specificity: Reductive cleavage C-S bond	
N/A because it requires strict maintenance under anaerobic conditions	( Malani *et al*., 2021)
Oxidative cleavage C-S bond, 4S pathway	
*Actinomycetales*	( El-Gendy and Nassar, 2018)
*Achromobacter*	( Bordoloi *et al*., 2014)
*Agrobacterium strain*	
*Arthrobacter*	( Ismail *et al*., 2016)
*Anaerobic* microorganism	
*Bacillus*	(( El-Gendy and Nassar, 2018), ( Nezammahalleh, 2015)
*Brevibacteria*	( El-Gendy and Nassar, 2018), ( Nassar *et al*., 2013), ( Nassar *et al*., 2016)
*Candida*	
*Corynebacteria*	
*Desulfobacterium aniline*	
*Desulfovibrio desulfuricans*	
*Gordonia* sp.	( El-Gendy and Nassar, 2018)
*Nocardia* sp.	
*Gordonia alkanivorans RIPI90A*	
*Klebsiella*	( El-Gendy and Nassar, 2018), ( Ismail *et al*., 2016)
*Microbacterium*	( El-Gendy and Nassar, 2018)
*Mycobacterium strains (G3y, phlei WU-F1, phlei WU- 0103*	( Ismail *et al*., 2016)
*Nocardia* sp.	
*Paenibacillus*	( El-Gendy and Nassar, 2018)
*Pseudomonas*	( Ismail *et al*., 2016)
*Rhodococcus erythropolis strains*	( El-Gendy and Nassar, 2018), ( Yaqoub, 2013), ( Shahaby and Essam-El-din, 2017), ( Alkhalili *et al*., 2017), ( El-Gendy *et al*., 2014)
*Rubio*	( El-Gendy and Nassar, 2018)
*Sphingomonas subarctica T7b*	( El-Gendy and Nassar, 2018), ( Gunam *et al*., 2021b)
*Stenotrophomonas sp. strain SA21*	( El-Gendy and Nassar, 2018), ( Mohamed *et al*., 2015), ( Ismail *et al*., 2016)
*Bacillus subrilis*	
*Bacillus strain Al 1-2*	
*Mycobacterium* sp.	
*Ps. aeruginosa*	( AL-Faraas *et al*., 2015)
*Paenibacillus* sp.	
Oxidative cleavage C-S bond, 2HBP sulfate end product pathway	
*Corynebacteria* sp.	
Oxidative cleavage C-S bond, 2HBP+ sulfate endproducts pathway	

Van Afferden demonstrated the differences between BDS and biodegradation (BDG) ( Van Afferden *et al*., 1990). The Konami kinetic model’s biodegradation mechanism can be compared to BDS, as illustrated in Figure 1. Some strains can be used in BDS, while others in BDG of oils ( Bahmani *et al*., 2018; Hamidi *et al*., 2021a,b).

The 4S pathway for biodesulfurization can be represented by the following set of equations:

1-
$\text{Dibenzothiophene}\;\left(\mathrm{DBT}\right)+\mathrm{ATP}+H_{2}O\to \mathrm{DBT}\;\text{monooxygenase}\to \mathrm{DBT}\;\text{sulfone}+\mathrm{AMP}+\mathrm{PPi}$

2-
$\mathrm{DBT}\;\text{sulfone}\to \text{desulfinase}\to 2‐\text{hydroxybiphenyl}\;\left(\mathrm{HBP}\right)+{{\mathrm{SO}}_{4}}^{2-}$

3-
$\mathrm{HBP}+\text{NADH}+H^{+}\to \mathrm{HBP}\;\text{dioxygenase}\to 2^{\prime }‐\text{hydroxybiphenyl}‐2‐\text{sulfinate}\;\left(\text{HBPS}\right)$

4-
$\text{HBPS}+H_{2}O\to \text{HBPS sulfatase}\to \text{biphenyl}‐2‐\text{sulfinate}\;\left(\mathrm{BPS}\right)+{{\mathrm{HSO}}_{4}}^{-}$

On the other hand, the Kodama pathway for biodegradation can be represented by the following set of equations:

1-
$\text{Dibenzothiophene}\;\left(\mathrm{DBT}\right)+O_{2}\to \mathrm{DBT}\;\text{dioxygenase}\to \mathrm{DBT}\;\text{sulfone}$

2-
$\mathrm{DBT}\;\text{sulfone}\to \text{desulfinase}\to 2‐\text{hydroxybiphenyl}\;\left(\mathrm{HBP}\right)+{{\mathrm{SO}}_{4}}^{2-}$

3-
$\mathrm{HBP}+O_{2}\to \mathrm{HBP}\;\text{dioxygenase}‐2‐\text{hydroxy}‐2^{\prime }‐\text{oxobiphenyl}‐3‐\text{sulfonate}\;\left(\text{HOBS}\right)$

4-
$\text{HOBS}+H_{2}O\to \text{HOBS sulfatase}\to 2‐\text{hydroxy}‐2^{\prime }‐\text{oxobiphenyl}‐3‐\text{sulfonate lactone}\;\left(\text{HOBL}\right)+{{\mathrm{HSO}}_{4}}^{-}$

5-
$\text{HOBL}+H_{2}O\to \text{HOBL hydrolase}‐2‐\text{hydroxy}‐2^{\prime }‐\text{oxobiphenyl}‐3‐\text{carboxylate}\;\left(\text{HOCB}\right)$

6-
$\text{HOCB}+O_{2}\to \text{HOCB dioxygenase}‐2‐\text{hydroxy}‐6‐\mathrm{oxo}‐6‐\text{phenylhexa}‐2,4‐\text{dienoate}\;\left(\text{HOPDA}\right)+{\mathrm{CO}}_{2}$

7-
$\text{HOPDA}+H_{2}O\to \text{HOPDA hydrolase}‐2‐\text{hydroxy}‐6‐\mathrm{oxo}‐6‐\text{phenylhexa}‐2,4‐\text{dienoic acid}\;\left(\text{HOPDA acid}\right)$

### 7.2 Mathematical models, simulation, and optimization

Limited efforts have been made to engineer BDS due to the lack of commercialization ( Malani *et al*., 2021). Various models have been developed to understand the BDS phenomenon, which can be classified based on the pretreated fluid (single or multiple substrates) or the system or phenomena design-based models ( Abin-Fuentes *et al*., 2014; Maass *et al*., 2015; Zhang *et al*., 2013). Metabolism-based models ( Bhatia and Sharma, 2012; Calzada *et al*., 2012) can also be used when considering a single substrate. Gross mathematical models, such as Monod, Haldane, or similar models, are commonly used for preliminary evaluations and to compare microbial cultures’ desulfurization efficiencies. These models need to be improved to consider the interaction between competition and inhibition ( Dejaloud *et al*., 2019; Irani *et al*., 2011). A study by Kareem in 2013 modeled the BDS of real kerosene in a batch reactor ( Kareem *et al*., 2013).

## 8. Qualification for industrialization and commercialization applicability

Efforts to engineer BDS have been limited, as it has not yet been commercialized ( Malani *et al*., 2021). To achieve the required desulfurization rate and efficiency without side effects, both experimental and theoretical development are needed. This includes rapid isolation and bio-identification characterization, as well as reaching high efficiency and rate in thermotolerant conditions using available chemicals such as solvents, and reducing product toxicity. Additionally, reusability and stability of the biocatalyst are important criteria ( Alkhalili *et al*., 2017; El-Gendy and Nassar, 2018). The reaction rate needs to be increased by 500-fold ( Malani *et al*., 2021; Nassar *et al*., 2016), and minimizing OWR is crucial to reduce separation difficulties. Mathematical models such as Monod, Haldan, or similar models are useful for preliminary evaluation and comparing microbial cultures’ desulfurization efficiencies. However, these models need to consider the interaction between competence and inhibition ( Dejaloud *et al*., 2019; Irani *et al*., 2011). Kareem (2013) studied the modeling of BDS of real kerosene in a batch reactor.

## 9. Summary of main points

• Fossil fuels’ sulfur content poses a significant challenge in meeting quality, health, safety, and environmental regulations.
• BDS is a desulfurization process used for removing sulfur from fuels and crude oil.
• The development of microorganism-based bioreactors is crucial for BDS.

## Data Availability

No data are associated with this article.
