# Supplementary material for: Biodesulfurization of Fossil Fuels: Analysis and Prospective
Source: F1000Res. 2023 Sep 8;12:1116. [Version 1] doi: 10.12688/f1000research.133427.1 (PMC10964007; doi:10.12688/f1000research.133427.1)
Supplement: Peer review history

# Reviewer report: Reviewer response for version 1

**DOI:** 10.5256/f1000research.146416.r252876

**Published:** 2024-05-08

**Authors:** Jasim I. Humadi

**Affiliations:** 1 Petroleum and Gas Refining Engineering Department, Tikrit University, Tikrit, Saladin Governorate, Iraq

The topic of this paper interested. It is observed goodly. However, It should incorporate the following corrections:

 1) The abstract must be written more clear about the important highlights in bio desulfurization technology

 2) In the introduction, the authors mentioned that the lest ppmw of sulfur is 15 in diesel fuel. What is the reference. The update level for sulfur content based on EPA in zero.

 3)The benefits of bio desulfurization over other technologies should be provided in separated section

 4)The essential bio catalysts for this technology must be observed clearly

 5) The language must be enhanced

 6) Add suggested mechanism as a figure based on the previous works

 7) The conclusion must be improved. Mention more highlights about the technology

 Enhancement of Desulfurization Process for Light Gas Oil Using New Zinc Oxide Loaded Over Alumina Nanocatalyst

 Mathematical model, simulation and scale up of batch reactor used in oxidative desulfurization of kerosene[6],[7],[8].

 F, Mohammed, et.al., 2022(Ref 9)

 I, Jasim, et.al., 2023 (Ref 10)

 I only recommend the authors to enhance the paper by more new related references. I don't say "must be" but I say "can".

Is the review written in accessible language?

Partly

Are all factual statements correct and adequately supported by citations?

Yes

Are the conclusions drawn appropriate in the context of the current research literature?

Yes

Is the topic of the review discussed comprehensively in the context of the current literature?

Yes

Reviewer Expertise:

Chemical engineering

I confirm that I have read this submission and believe that I have an appropriate level of expertise to confirm that it is of an acceptable scientific standard.

## References

[1] : Dimensionless evaluation and kinetics of rapid and ultradeep desulfurization of diesel fuel in an oscillatory baffled reactor. *RSC Adv* .2022;12(23) : 10.1039/d2ra01663j 14385-14396 10.1039/d2ra01663j 35733914 PMC9149686

[2] : Evaluation the performance of the tin (IV) oxide (SnO2 ) in the removal of sulfur compounds via oxidative-extractive desulfurization process for production an eco-friendly fuel. *International Journal of Chemical Reactor Engineering* .2023;21(6) : 10.1515/ijcre-2022-0046 727-741 10.1515/ijcre-2022-0046

[3] : Improving the extraction of sulfur-containing compounds from fuel using surfactant material in a digital baffle reactor. *Materials Today: Proceedings* .2021;42: 10.1016/j.matpr.2020.11.821 1777-1783 10.1016/j.matpr.2020.11.821

[4] : Design of new nano-catalysts and digital basket reactor for oxidative desulfurization of fuel: Experiments and modelling. *Chemical Engineering Research and Design* .2023;190: 10.1016/j.cherd.2022.12.043 634-650 10.1016/j.cherd.2022.12.043

[5] : Fast, non-extractive, and ultradeep desulfurization of diesel in an oscillatory baffled reactor. *Process Safety and Environmental Protection* .2021;152: 10.1016/j.psep.2021.05.028 178-187 10.1016/j.psep.2021.05.028

[6] : Efficient immobilization of acids into activated carbon for high durability and continuous desulfurization of diesel fuel. *Energy Science & Engineering* .2023;11(10) : 10.1002/ese3.1545 3662-3679 10.1002/ese3.1545

[7] : Process modeling and kinetic estimation for desulfurization of diesel fuel using nano - ZnO/Al2O3. *Chemical Industry and Chemical Engineering Quarterly* .2024;30(2) : 10.2298/CICEQ230208020H 151-159 10.2298/CICEQ230208020H

[8] : Improvement of extractive desulfurization for Iraqi refinery atmospheric residual. *Petroleum Science and Technology* .2024; 10.1080/10916466.2023.2292784 1-14 10.1080/10916466.2023.2292784

[9] : Improvement of design synthetic nano-catalysts for performance enhancement of oxidative desulfurization using batch reactor. *ResearchGate* .2022; 10.1063/5.0109089 10.1063/5.0109089

[10] : Improved Kerosene Quality with the Use of a Gamma Alumina Nanoparticles Supported Zinc Oxide Catalyst in a Digital Batch Baffled Reactor: Experiments and Process Modelling.2023;226-237

# Reviewer report: Reviewer response for version 1

**DOI:** 10.5256/f1000research.146416.r241257

**Published:** 2024-03-28

**Authors:** Jaykumar Bhasarkar

**Affiliations:** 1 Laxminarayan Institute of Technology, Nagpur, Maharashtra, India

The manuscript entitled "Biodesulfurization of Fossil Fuels: Analysis and Prospective"provide the insight into the biodesulfurization of fossil fuel, however, some modification can improve the quality of the paper. 

 1. In introduction part, it is recommended to give more information about the overall desulfruization process such as conventional process (hydrodesulfruization). Also it required to give brief about all thee other alternative methods for the desulfurization of fossil fuels. 

 2.In present manuscript, authors have not mention about the metabolic pathways followed by  microorganisms. Therefore, it is essential to provide the metabolic pathway such as 4C pathway and Kodoma pathway with their merits and demerits. 

 3.Some more literature needs to be added to the present manuscript : [Ref 1,2,3,4]

 4.In modeling section, authors are requested to provide more details about the kinetic model and inhibition models of the cell growth and substrate consumption. [refer : Ref 1].

 The manuscript is mainly focused on a detailed review on bio desulfurization process. The present manuscript cover good-quality information on biodesulfurization process but it requires to provide merits and demerits of the other desulfurization processes.Therefore, some more alternative technologies need to be addressed in the present manuscript. It is essential to cite more references on alternative methodologies adopted for efficient desulfurization. It is also requested to provide the comparative table for the biodesulfurization process.

Is the review written in accessible language?

Yes

Are all factual statements correct and adequately supported by citations?

Partly

Are the conclusions drawn appropriate in the context of the current research literature?

Partly

Is the topic of the review discussed comprehensively in the context of the current literature?

Yes

Reviewer Expertise:

Nanomaterials, Desulfruization of liquid fuels, sonochemistry

I confirm that I have read this submission and believe that I have an appropriate level of expertise to confirm that it is of an acceptable scientific standard, however I have significant reservations, as outlined above.

## References

[1] : Kinetic modelling and process engineering aspects of biodesulfurization of liquid fuels: Review and analysis. *Bioresource Technology Reports* .2021;14: 10.1016/j.biteb.2021.100668 10.1016/j.biteb.2021.100668

[2] : Ultrasound assisted biodesulfurization of liquid fuel using free and immobilized cells of Rhodococcus rhodochrous MTCC 3552: A mechanistic investigation. *Bioresour Technol* .2015;187: 10.1016/j.biortech.2015.03.102 369-378 10.1016/j.biortech.2015.03.102 25863901

[3] : Physical insight into ultrasound-assisted biodesulfurization using free and immobilized cells of Rhodococcus rhodochrous MTCC 3552. *Chemical Engineering Journal* .2016;295: 10.1016/j.cej.2016.03.042 254-267 10.1016/j.cej.2016.03.042

[4] : Mechanistic analysis of ultrasound assisted enzymatic desulfurization of liquid fuels using horseradish peroxidase. *Bioresour Technol* .2015;196: 10.1016/j.biortech.2015.07.063 88-98 10.1016/j.biortech.2015.07.063 26231128

# Reviewer report: Reviewer response for version 1

**DOI:** 10.5256/f1000research.146416.r227751

**Published:** 2024-03-25

**Authors:** Talib M. Albayati

**Affiliations:** 1 University of Technology, Karrada, Iraq

**1.** I have not found comparative study with other studies in this field.

**2.** Re-write the introduction part so as to give more details and mention the sulfur effect on environment.

**3. ** In introduction give more emphasis to the fact that most of the previous research refers to petroleum products rather than crude oil. 

**4. ** The aims of the present work must be more cleared at the end of the introduction part. 

**5. ** What is the mechanism of Ionic Liquids aqueous solution for sulfur removal from actual crude oil. 

**6. **The Ionic Liquids aqueous solution surface area is very important. The author must be discussing this point in detail because the surface area is very important factor increasing the removal rate.

**7.** What is the role of Ionic Liquids aqueous solution? Is it extraction solution or catalytic solution? How we can guess which one is the working?

**8.** What about the properties of Fuel desulfurization after treatment or removal sulfur? 

**9.** I hope from the author to draw figure explain the relationship between the actual and predict values (Residual plots for the desulfurization).

**10.** What is the main conclusion from this study? The conclusion must be reduced.

**11.** Clarify the term "Sulfur wt %" (I assume it is the percentage of sulfur in the product oil).

**12.** I hope from the author of this paper to give more details about optimization method with 3D figure for the result of this paper.

Is the review written in accessible language?

Yes

Are all factual statements correct and adequately supported by citations?

Yes

Are the conclusions drawn appropriate in the context of the current research literature?

Yes

Is the topic of the review discussed comprehensively in the context of the current literature?

Yes

Reviewer Expertise:

Chemical Engineering

I confirm that I have read this submission and believe that I have an appropriate level of expertise to confirm that it is of an acceptable scientific standard, however I have significant reservations, as outlined above.

# Reviewer report: Reviewer response for version 1

**DOI:** 10.5256/f1000research.146416.r252874

**Published:** 2024-03-21

**Authors:** Iqrash Shafiq

**Affiliations:** 1 Department of Chemical Engineering, COMSATS University Islamabad, Islamabad, Islamabad Capital Territory, Pakistan

The provided text appears to be an in-depth review article on biodesulfurization (BDS) of fossil fuels. Here's a summary of the key points covered in each section:

1. **Abstract**: The abstract provides an overview of BDS as a promising method for treating sulfur in fossil fuels, highlighting its environmental benefits and technical efficiency.
2. **Introduction**: This section introduces the importance of fossil fuels, the need for sulfur treatment due to health, safety, and environmental concerns, and the regulations surrounding sulfur content in fuels.
3. **Desulfurization methods**: Various methods of desulfurization are discussed, including physical, chemical, and biological methods. BDS is emphasized for its efficiency and cost-effectiveness compared to other methods like hydrodesulfurization (HDS).
4. **Susceptibility of treated oils and biodesulfurizers**: Different microorganisms and their applications in BDS for treating various fractions of crude oil and model compounds are discussed.
5. **Biodesulfurization performance**: Factors affecting BDS efficiency, such as physical, chemical, and biological factors, are explored. The role of microorganisms, environmental conditions, and biocatalysts in BDS efficiency is highlighted.
6. **Experimental developments techniques**: This section covers experimental techniques and developments in BDS, including biocatalysts, reactor systems, environmental factors, and optimization studies.
7. **Theoretical development methods**: Mathematical modeling and theoretical aspects of BDS are discussed, including kinetic mechanisms, metabolic pathways of microorganisms, and mathematical models for simulating the BDS process.

Overall, the article provides a comprehensive overview of BDS, covering its methods, performance, experimental developments, and theoretical aspects. It highlights the importance of BDS as a promising method for reducing sulfur content in fossil fuels, addressing environmental concerns and regulatory requirements.

Is the review written in accessible language?

Yes

Are all factual statements correct and adequately supported by citations?

Yes

Are the conclusions drawn appropriate in the context of the current research literature?

Yes

Is the topic of the review discussed comprehensively in the context of the current literature?

Yes

Reviewer Expertise:

Desulfurization; Photocatalysis

I confirm that I have read this submission and believe that I have an appropriate level of expertise to confirm that it is of an acceptable scientific standard.

# Reviewer report: Reviewer response for version 1

**DOI:** 10.5256/f1000research.146416.r205148

**Published:** 2023-10-09

**Authors:** Shuiquan Chen

**Affiliations:** 1 Shandong University of Science and Technology, Qingdao, Shandong, China

The article summarizes the main advantages and challenges of BDS, as well as the future research directions. The article points out that BDS is an environmentally friendly, low-cost, and high-efficiency method that can achieve ultra-low sulfur diesel (ULSD) standards, but it still needs to improve the reaction rate, biocatalyst stability and reusability, and engineering and commercialization efforts. I recommend accepting it with the following major changes that can complement the text content:

1. Some details need to be modified, such as “B.T., DBT, DBTO2, M DBTSO2, MgSO4, BNT, DBS, 2,8 DMDBT, 2,6 DNDBT, DMDBT, thianthrene, and dibenzyl sulfide ” in page 4.
2. Some abbreviations are not defined in the article: e.g.: BNT, 4,6-DB-DBT, 4-HDBT, etc. please check and correct them throughout the text.
3. It has been more than thirty years since the first biodesulfurization bacterium was discovered, but the biodesulfurization technology has not been industrialized yet. Please summarize the existing problems in detail.

Is the review written in accessible language?

Yes

Are all factual statements correct and adequately supported by citations?

Yes

Are the conclusions drawn appropriate in the context of the current research literature?

Yes

Is the topic of the review discussed comprehensively in the context of the current literature?

Yes

Reviewer Expertise:

Biodesulfurization; bioremediation

I confirm that I have read this submission and believe that I have an appropriate level of expertise to confirm that it is of an acceptable scientific standard, however I have significant reservations, as outlined above.

# Response

**Published:** 2023-10-09

**Authors:** Wisam Al-Khazaali

**Affiliations:** Chemical Engineering, Shahid Bahonar University of Kerman, Kerman, Kerman Province, Iran

Thank you for important notes. I am doing the note.

# Response

**Published:** 2024-02-22

**Authors:** Wisam Al-Khazaali

**Affiliations:** Chemical Engineering, Shahid Bahonar University of Kerman, Kerman, Kerman Province, Iran

I confirm that I finish these suggestions from reviewer. Thank you for your cooperation.
